# *In vivo* investigation of Lcr35^®^ anti-candidiasis properties in *Caenorhabditis elegans* reveals the involvement of highly conserved immune pathways

**DOI:** 10.3389/fmicb.2022.1062113

**Published:** 2022-12-23

**Authors:** Cyril Poupet, Étienne Rifa, Sébastien Theil, Muriel Bonnet, Philippe Veisseire, Guillaume Cardin, Élise Guéret, Stéphanie Rialle, Christophe Chassard, Adrien Nivoliez, Stéphanie Bornes

**Affiliations:** ^1^Université Clermont Auvergne, INRAE, VetAgro Sup, UMRF, Aurillac, France; ^2^MGX, Univ Montpellier, CNRS, INSERM, Montpellier, France; ^3^Biose^®^ Industrie, Aurillac, France

**Keywords:** *Caenorhabditis elegans*, probiotic, LBM, candidiasis, host response, mechanisms of action

## Abstract

Lactic acid bacteria, including the microorganisms formerly designated as *Lactobacillus*, are the major representatives of Live Biotherapeutic Microorganisms (LBM) when used for therapeutic purposes. However, in most cases, the mechanisms of action remain unknown. The antifungal potential of LBM has already been demonstrated using preclinical models (cell cultures, laboratory animals). Understanding their mechanisms of action is strategic for the development of new therapeutics for humans. Here, *Caenorhabditis elegans* was used as an *in vivo* model to analyze pro-longevity, anti-aging and anti-candidiasis effects of the LBM *Lacticaseibacillus rhamnosus* (formerly *Lactobacillus rhamnosus*) Lcr35^®^. A high-throughput transcriptomic analysis revealed a specific response of *C. elegans* depending on whether it is in the presence of the LBM *L. rhamnosus* Lcr35^®^ (structural response), the yeast *Candida albicans* (metabolic response) or both (structural and metabolic responses) in a preventive and a curative conditions. Studies on *C. elegans* mutants demonstrated that the p38 MAPK (*sek-1*, *skn-1*) and the insulin-like (*daf-2*, *daf-16*) signaling pathways were involved in the extended lifespan provided by *L. rhamnosus* Lcr35^®^ strain whereas the JNK pathway was not involved *(jnk-1)*. In addition, the anti *C. albicans* effect of the bacterium requires the *daf-16* and *sek-1* genes while it is independent of *daf-2* and *skn-1*. Moreover, the anti-aging effect of Lcr35^®^, linked to the extension of longevity, is not due to protection against oxidative stress (H_2_O_2_). Taken together, these results formally show the involvement of the p38 MAP kinase and insulin-like signaling pathways for the longevity extension and anti-*Candida albicans* properties of Lcr35^®^ with, however, differences in the genes involved. Overall, these findings provide new insight for understanding the mechanisms of action of a probiotic strain with antimicrobial potential.

## 1. Introduction

Microorganisms with probiotic potential are especially known for their role in gut homeostasis (van Baarlen et al., 2013) and in enhancing host immunity (Lazar et al., 2018). Initially defined by the World Health Organization (WHO) and the Food and Agriculture Organization of the United Nations (FAO) as ‘live microorganisms, which, when administered in adequate amounts, confer a health benefit on the host’ (FAO and WHO, 2001), these have traditionally been isolated from the commensal microbiota of human gastrointestinal tract (Sornplang and Piyadeatsoontorn, 2016) or from fermented products such as dairy products (Mohammed and Çon, 2021). From a regulatory standpoint, their harmlessness to humans has enabled them to be considered as Generally Regarded as Safe (GRAS) by the United States Food and Drug Administration (FDA) or as Qualified Presumption of Safety (QPS) by the European Food Safety Authority (EFSA; Martín and Langella, 2019). With the expansion of molecular methods for the diagnosis and characterization of pathological disorders, the importance of microbiota balance in a host in promoting health has become evident. Therefore, innovative therapeutic practices have been developed such as the use of living microorganisms to restore a satisfactory microbiota balance. The American and European regulatory authorities, *via* the FDA and the European Pharmacopeia have recently designated these new medicinal products as Live Biotherapeutic Products (LBPs; Rouanet et al., 2020). An LBP, as defined by the FDA Center for Biologics Evaluation and Research (CBER), is ‘a biological product that: (1) contains live organisms, such as bacteria; (2) is applicable to the prevention, treatment, or cure of a disease or condition of human beings; and (3) is not a vaccine’ (Dreher-Lesnick et al., 2017). On the other hand, the European Pharmacopeia defines them as “medicinal products containing live microorganisms (bacteria or yeasts) for human use” (Rouanet et al., 2020).

The first investigations performed on lactic acid–producing bacteria (LAB) and their health effects in humans by Metchikoff (1908) suggested that their ingestion improved host health. Most studies concerning probiotic LABs have shown their beneficial effects using *in vitro* models but lack insight into the potential mechanism of action (Metchikoff, 1908).

*Caenorhabditis elegans* is a small soil nematode that primarily feeds on bacteria. It is widely used as an experimental system for various biological research studies including aging, bacterial infection, and immunological response, because of their morphological simplicity, suitability for genetic analysis, ease of maintenance in the laboratory, and short lifespan and reproductive cycle (Meneely et al., 2019; Poupet et al., 2019a,b).

Due to its genetic and protein proximity to humans (Lai et al., 2000), the *C. elegans* model allows experiments to be carried out in order to characterize the interactions between different microorganisms and a host. It has already been used to study the pathogenicity mechanisms of *Candida albicans* (Elkabti et al., 2018; Feistel et al., 2019). Pukkila-Worley and colleagues discovered and characterized the antifungal response put in place by the worm upon contact with the yeast. This response involves the expression of genes encoding for antifungal immune factors (*fipr-22*, *fipr-23*) but also detoxification enzymes (*trx-3*). They also demonstrated that part of these antimicrobials was dependent on the p38 MAPK signaling pathway, a pathway that is highly conserved in the animal kingdom (Pukkila-Worley et al., 2011). However, few studies have been conducted with the nematode on the use of probiotic microorganisms for the treatment of *C. albicans* fungal infection (de Barros et al., 2017; Poupet et al., 2019a,b,c).

Precedent *in vitro* investigations of Nivoliez et al. (2012) revealed a high killing rate of *C. albicans* induced by *Lacticaseibacillus rhamnosus* (formerly *Lactobacillus rhamnosus*) Lcr35^®^. The authors demonstrated that although *L. rhamnosus* Lcr35^®^ adheres little to epithelial cells, it favorably allows the restoration of commensal microbiota (Petricevic and Witt, 2008; Yeom et al., 2015). Until now, the molecular mechanisms responsible for the anti-pathogenic activity of *L. rhamnosus* Lcr35^®^ have been little described in the literature. The work of Dausset et al. (2020) was interested in the mechanisms of action of Gynophilus, a Live Biotherapetic Product (LBP) containing *L. rhamnosus* Lcr35^®^ and sodium thiosulfate, an excipient of the product which potentiate the anti-*Candida* activity. Thanks to a volatolomic analysis, the authors thus managed to demonstrate the production of a sulfur metabolite, S-methylthioacetate, with fungicidal activity and whose synthesis by *L. rhamnosus* Lcr35^®^ is strongly induced by sodium thiosulfate (Dausset et al., 2020). Although reaching a milestone in the understanding of the mechanism of action, these results did not allow for the moment to describe the molecular anti *Candida albicans* mechanisms set up by the native strain of *L. rhamnosus* Lcr35^®^. The establishment of animal models sufficiently close to humans is therefore necessary in order to characterize them.

In our previous studies, the probiotic properties of *L. rhamnosus* Lcr35^®^ were highlighted in the nematode *C. elegans* model. We have demonstrated that *L. rhamnosus* Lcr35^®^ induces a significant increase in the longevity of the nematode when it was fed with this bacterium. We hypothesized that this increase in longevity was related to activation of the transcription factor FOXO DAF-16. On the other hand, we did not note any impact of the bacterial strain on the growth of the nematode (Poupet et al., 2019c). When we simulated a preventive or a curative approach using probiotic against fungal infection with *Candida albicans* in *C. elegans*, *L. rhamnosus* Lcr35^®^ provided effective protection of the host against candidiasis. A significant increase in longevity has been shown in these fungal infection conditions, although the presence of yeast in the digestive tract of the worm has been detected (Poupet et al., 2019a,b,c). Using preliminary mechanistic analysis, we hypothesized that preventive therapy appeared to suppress the immune response of the nematode with repression of genes encoding antimicrobial peptides as well as the p38 MAPK pathway, suggesting the yeast no longer appeared to be detected by the worm immune system. Conversely, during a curative treatment, we observed the stimulation of host immunity. These two works contributed to demonstrate the relevance of the nematode model to study the interactions between different microorganisms but also with a host, here between a bacterium, a yeast and a metazoan.

In this paper, we focused on the study of the mechanisms of action allowing explaining the preventive probiotic properties of *L. rhamnosus* Lcr35^®^ including the pro-survival and anti-*Candida albicans* activities. For this, the invertebrate model *Caenorhabditis elegans* was used to characterize the signaling pathways of the host involved in the response to the ingestion of a probiotic strain and/or a pathogenic *C. albicans* strain. This response was assessed by conducting a global transcriptomic analysis by high throughput sequencing of messenger RNAs from *C. elegans* after probiotic or *C. albicans* ingestion or in prophylactic and curative conditions. This analysis was completed by the study of longevity and survival to the infection of mutant worms to identify key genes necessary for *L. rhamnosus* Lcr35^®^ probiotic effects. Also, we challenged *C. elegans* with a hydrogen peroxide treatment in order to determine if *L. rhamnosus* Lcr35^®^ provide a protection to the host. Indeed, some authors showed a causal relationship between probiotics, oxidative stress and longevity (Grompone et al., 2012; Senchuk et al., 2018). The works presented here represent the first study highlighting the anti *C. albicans* mechanisms of action of a *Lacticaseibacillus* using the invertebrate model *C. elegans*. Also, no vertebrate animals were used for experimental purposes.

## 2. Materials and methods

### 2.1. Microbial strains and growth conditions

The *E. coli* OP50 strain was provided by the *Caenorhabditis* Genetics Center (Minneapolis, MN, United States) and was grown on Lysogeny Broth (LB, Miller’s Modification) (Conda, Madrid, Spain) at 37°C overnight. The *L. rhamnosus* Lcr35^®^ strain was provided by biose^®^ Industrie (Aurillac, France) and was grown in de Man, Rogosa, Sharpe (MRS) broth (bioMérieux, Marcy l’Etoile, France) at 37°C overnight. *C. albicans* ATCC^®^ 10231™ was grown in yeast peptone glucose (YPG) broth pH 6.5 (per L: 10 g yeast extract, 10 g peptone, 20 g glucose) at 37°C for 48 h. Microbial suspensions were spun down for 15 min at 4,000 rpm (Rotofix 32A, Hettich Zentrifugen, Tuttlingen, Germany) and washed with M9 buffer (per L: 3 g KH_2_PO_4_, 6 g Na_2_HPO_4_, 5 g NaCl, 1 ml 1 M MgSO_4_) to obtain a final concentration of 100 mg/ml.

### 2.2. *Caenorhabditis elegans* maintenance

*Caenorhabditis elegans* N2 (wild-type), CB1370 (*daf-2*(e1370) III), GR1307 (*daf-16*(mgDf50) I), KU4 (*sek-1*(km4) X), LG333 (*skn-1*(zu135) IV; gels7) and VC8 (*jnk-1*(gk7) IV) strains were acquired from the *Caenorhabditis* Genetics Center. The nematodes were grown and maintained at 20°C (15°C for the CB1370 strain) on nematode growth medium (NGM) (per L: 3 g NaCl; 2.5 g peptone; 17 g agar; 5 mg cholesterol; 0.11 g CaCl_2_; 0.12 g MgSO_4_, 25 ml 1 M potassium phosphate buffer at pH 6) plates supplemented with yeast extract (4 g/l) (NGMY) and seeded with *E. coli* OP50 (Brenner, 1974).

### 2.3. *Caenorhabditis elegans* synchronization

To avoid variations in results due to age differences, a worm synchronous population was required. Gravid worms were washed off using M9 buffer and spun down for 2 min at 1,500 rpm. Five milliliters of worm bleach (2.5 ml of M9 buffer, 1.5 ml of bleach (2.6°Chl), 1 ml of 5 M sodium hydroxide) were added to the pellet and vigorously shaken until adult worm body disruption. The action of worm bleach was stopped by adding 20 ml of M9 buffer. The egg suspension was then spun down for 2 min at 1,500 rpm and washed twice with 20 ml of M9 buffer. Eggs were allowed to hatch under slow agitation at 25°C for 24 h in approximately 20 ml of M9 buffer. L1 larvae were then transferred onto NGMY plates seeded with *E. coli* OP50 until they reached the L4/young adult stage.

### 2.4. Oxidative stress assay on agar plates

The aim is to characterize the potential antioxidant activity of the *L. rhamnosus* Lcr35^®^ probiotic strain on *C. elegans* N2 (wild-type) as described by Cardin et al. (2021). Briefly, 100 microliters of *L. rhamnosus* Lcr35^®^ or *E. coli* OP50 suspension (100 mg/ml) were spread on peptone-free NGM containing 0.12 mM 5-fluorodeoxyuridine (FUdR) (Sigma, Saint-Louis, United States) (6-well plate) and incubated at 37°C overnight. Synchronized L4/young adult worms were transferred onto each well and incubated at 20°C for 5 days and then transferred onto either peptone-free NGM (control) or peptone-free NGM with 3 mM H_2_O_2_ (Acros organics, Geel, Belgium). After 3.5 h of incubation, worm viability was scored and the survival rate σ_r_ was calculated using the following formula:


σr=(N3.5N0)withH2O2(N3.5N0)withoutH2O2

with:

– *σ*_r_: survival rate
– N_0_: number of alive worms at *t* = 0 h
– N_3.5_: number of alive worms at *t* = 3.5 h

An animal was scored as dead when it did not respond to a gentle mechanical stimulation. This assay was performed as three independent experiments with three plates per condition. Differences between conditions were determined by an unpaired *t* test using GraphPad Prism version 9.1.1 for macOS. A value of *p* ≤ 0.05 was considered as significant.

### 2.5. Effect of bacterial and fungal strains on *Caenorhabditis elegans*

The workflow analysis of the effect of microbial strains on *C. elegans* is graphically represented in Supplementary Figure S1.

#### 2.5.1. Preparation of plates containing probiotic bacteria or pathogenic yeasts

One hundred microliters of *L. rhamnosus* Lcr35^®^ or *E. coli* OP50 suspension (100 mg/ml) were spread on NGMY +0.12 mM FUdR plates and incubated at 37°C overnight. Concerning *C. albicans* strains, 100 μl of the suspension were spread on Brain Heart Infusion BHI (Biokar Diagnostics, Beauvais, France) + 0.12 mM FUdR plates and incubated at 37°C overnight.

#### 2.5.2. *Caenorhabditis elegans* lifespan assay

Synchronous L4 worms were transferred [~50 worms per well (6-well plate)] to NGMY with 0.12 mM FUdR to avoid egg hatching and seeded with 100 μl of microbes at 100 mg/ml *E. coli* OP50, *L. rhamnosus* Lcr35^®^ or *C. albicans* as previously stated. The plates were kept at 20°C (15°C for the CB1370 strain), and live worms were scored each day until the death of all animals. An animal was scored as dead when it did not respond to a gentle mechanical stimulation. This assay was performed as three independent experiments with three plates per condition.

The *C. elegans* lifespan assay was examined using the Kaplan–Meier method, and differences were determined using the log-rank test with R software version 3.6.1 (R Core Team, 2018), and the *survival* (Therneau, 2015) and *survminer* (Kassambara and Kosinski, 2017) packages. A value of *p* ≤ 0.05 was considered significant.

#### 2.5.3. Effects of *Lacticaseibacillus rhamnosus* Lcr35^®^ on candidiasis in *Caenorhabditis elegans*: Preventive and curative treatment survival assays

Sequential feeding with *L. rhamnosus* Lcr35^®^ and *C. albicans* was induced in *C. elegans* in all experiments. As control groups, monotypic contamination was induced in *C. elegans* by inoculation with only *C. albicans*, *L. rhamnosus* Lcr35^®^ or *E. coli* OP50.

The survival assay was performed according to the work of de Barros et al. (2017), with some modifications. During a preventive treatment, young adult worms were placed on plates containing *L. rhamnosus* Lcr35^®^ at 20°C for 4 h. Next, the worms were washed with M9 buffer to remove bacteria prior to being placed on *C. albicans* plates for 2 h at 20°C. Infected nematodes were washed off plates using M9 buffer prior to being transferred to a 6-well microtiter plate (approximately 50 worms per well) containing 2 ml of BHI/M9 (20%/80%) with 0.12 mM FUdR liquid assay medium per well and incubated at 20°C (15°C for the CB1370 strain). During a curative treatment, young adult worms were placed on plates containing *C. albicans* at 20°C for 2 h and 4 h with *L. rhamnosus* Lcr35^®^ at 20°C after washing. For the control groups (i.e., *E. coli* OP50 + *C. albicans*, *E. coli* OP50 only, *L. rhamnosus* Lcr35^®^ only and *C. albicans* only), worms were treated in the same way. Nematodes were observed daily and were considered dead when they did not respond to a gentle mechanical stimulation. This assay was performed as three independent experiments containing three wells per condition.

The *C. elegans* survival assay was examined using the Kaplan–Meier method, and differences were determined using the log-rank test with R software version 3.6.1 (R Core Team, 2018), and the *survival* (Therneau, 2015) and *survminer* (Kassambara and Kosinski, 2017) packages. A value of *p* ≤ 0.05 was considered significant.

### 2.6. *Caenorhabditis elegans* whole transcriptome analysis

#### 2.6.1. Sample preparation for RNA analysis

Worms were fed either with a single strain (*E. coli* OP50 or *L. rhamnosus* Lcr35^®^ or *C. albicans*) or two strains successively (*E. coli* OP50 or *L. rhamnosus* Lcr35^®^ first to *C. albicans*). Approximately 10,000 worms (N2 strain) were harvested from NGMY plates with M9 buffer. Total RNA was extracted by adding 500 μl of TRIzol reagent (Ambion by Life Technologies, Carlsbad, CA, United States). Worms were disrupted using a Precellys (Bertin Instruments, Montigny-le-Bretonneux, France) and glass beads (PowerBead Tubes Glass 0.1 mm, Mo Bio Laboratories, United States). Beads were removed by centrifugation at 14,000 rpm for 1 min (Eppendorf^®^ 5415D, Hamburg, Germany), and 100 μl of chloroform were added to the supernatant. Tubes were vortexed for 30 s and incubated at room temperature for 3 min. The phenolic phase was removed by centrifugation at 12,000 rpm for 15 min at 4°C. The aqueous phase was treated with chloroform as previously described. RNA was precipitated by adding 250 μl of isopropanol for 4 min at room temperature and spun down at 12,000 rpm for 10 min (4°C). The supernatant was discarded, and the pellet was washed with 1 ml of 70% ethanol. The supernatant was discarded after centrifugation at 14,000 rpm for 5 min (4°C), and the pellet was dissolved in 20 μl of RNase-free water.

Any DNA present in the samples was removed using the Invitrogen^™^ DNA-free^™^ DNA Removal Kit (Invitrogen, ref. AM1906, Carlsbad, CA, United States). Ten micrograms of RNA were mixed with 5 μl of 10X buffer, 0.5 μl of DNase and 35 μl of RNase-free water and incubated at 37°C for 30 min. Then, 0.5 μl of DNase were added and incubated again at 37°C for 30 min. Afterwards, DNase was inactivated by adding 5 μl of DNase Inactivation Reagent, mixed thoroughly and incubated for 2 min at room temperature. The samples were centrifuged for 2 min at 10,000 rpm (Beckman J2-MC, Beckman Coulter, Brea, CA, United States) and the supernatants were transferred into clean Eppendorf tubes.

The concentration of RNA in each sample was assessed with a Nanodrop 2000c spectrophotometer (Thermo Fisher Scientific, Wilmington, DE, United States) and the quality was evaluated using an Agilent 2,100 Bioanalyzer system (Agilent Technologies, Santa Clara, CA, United States). Samples were stored at −80°C until analysis.

#### 2.6.2. Determination of *Caenorhabditis elegans* transcriptome by RNA sequencing

The following steps were performed by the MGX Platform (Montpellier GenomiX, CNRS, Montpellier, France) using Illumina kits, devices and softwares (Illumina Inc., San Diego, CA, United States): construction of the mRNA library using a TruSeq Stranded mRNA Sample Preparation Kit; cluster generation with the cBot system using a Cluster Generation Kit; hybridization of the sequencing primer on the flow cell; 50-bp single-read sequencing using a HiSeq 2,500 device with Sequence by Synthesis technology; informatic pretreatments, i.e., image analysis with the HiSeq Control Software and Real-Time Analysis component, base-calling with the RTA software and demultiplexing with bcl2fastq. The data discussed in this publication have been deposited in NCBI’s Gene Expression Omnibus (Edgar et al., 2002) and accessible through GEO Series accession number GSE202877.^1^ The quality scores across all bases of all reads and the N (non-attributed bases) content across all bases were determined for each condition with the FastQC software from the Babraham Institute. The research of contaminants was carried out using FastQ Screen.

The reads were aligned on reference genomes using Bowtie2 software: *C. elegans* (WBcel235, UCSC), and *C. albicans* SC5314 Assembly 22 (Candida Genome Database). Reads which did not align on *C. elegans* genome were excluded from further analysis. Retained reads were counted using feature counts software (version 1.6.4) and analysis of differential expression was achieved by the package DESeq2 (Love et al., 2014). Gene expression changes with adjusted value of *p* of less than 0.05 were declared differentially expressed and considered for further analysis.

Using significantly differentially expressed genes (adjusted value of *p* less than 0.05), a Gene Ontology analysis was performed. For this, the genes were sorted according to their expression (under-expression or overexpression) and according to whether the worms were in contact with a single microbial strain or a sequential culture. The lists obtained were then analyzed using GO Enrichment Analysis. Three levels of analysis were considered: biological process, molecular function and cellular component. Only the GO terms significantly enriched (Fisher test and FDR, *p* < 0.05) were retained and represented graphically.

The representation of gene expression in the form of Volcano plots and GO terms were carried using GraphPad Prism version 9.1.1 for macOS. Clustering and heatmap representation were carried out using the Galaxy online platform (Afgan et al., 2018). Venn diagrams were created using the online tool Venny (Oliveros, n.d.).

## 3. Results

### 3.1. *Caenorhabditis elegans* loss-of-function mutants lifespan assay

**Figure 1:**
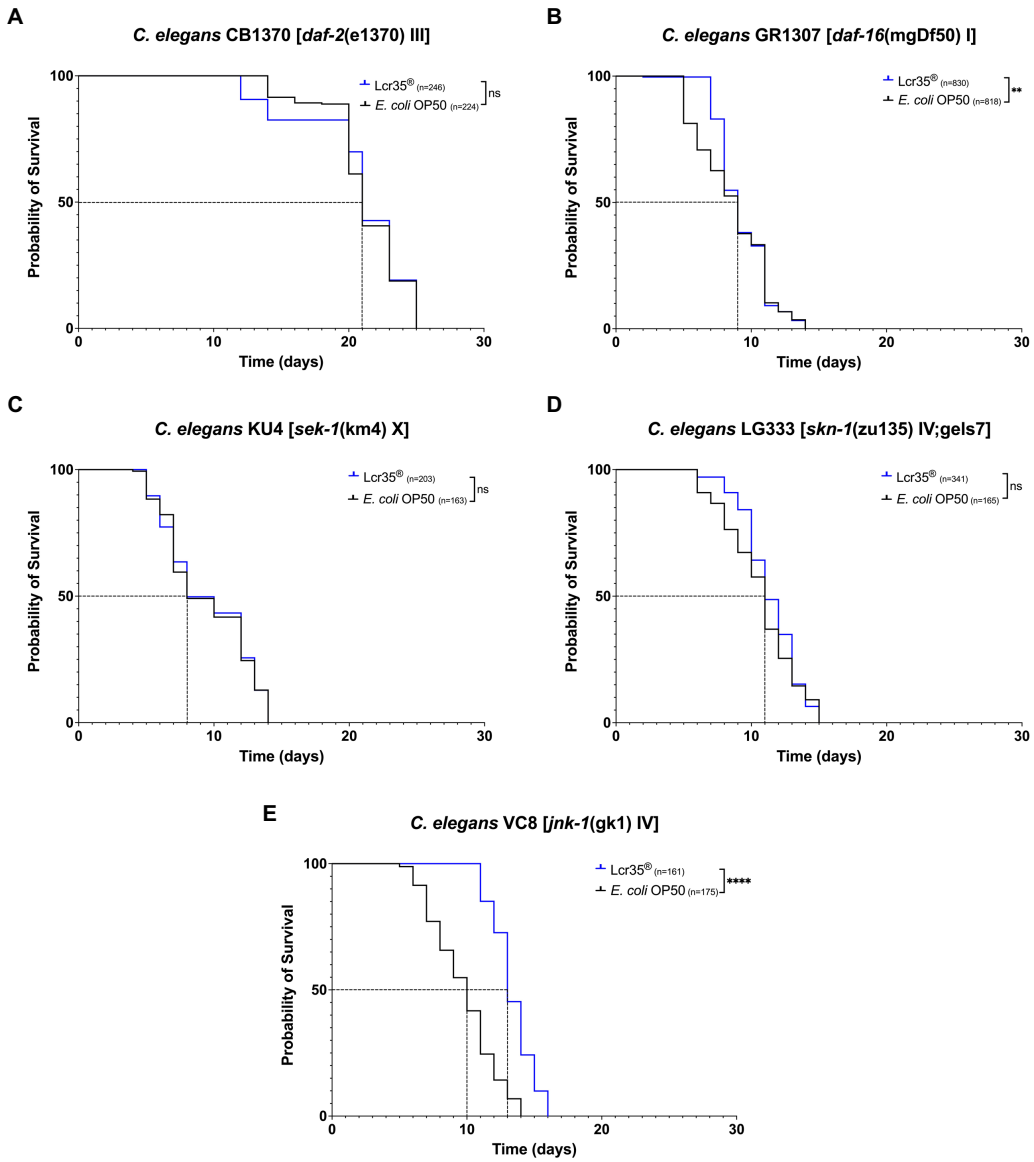
Influence of *Lactobacillus rhamnosus* Lcr35^®^ on the lifespan of *Caenorhabditis elegans daf-2* (CB1370) **(A)**, *daf-16* (GR1307) **(B)**, *sek-1* (KU4) **(C)**, *skn-1* (LG333) **(D)**, and *jnk-1* (VC8) **(E)** loss-of-function mutant strains. The mean lifespan, where half of the population was dead, is represented on the abscissa. The asterisks indicate the value of *p*s (log-rank test) with *E. coli* OP50 as a control (NS, not significant; **p < 0.01; ****p < 0.0001).

We investigated the effects of the probiotic *L. rhamnosus* Lcr35^®^ on lifespan of *C. elegans* loss-of-function mutant strains, in comparison of the *E. coli* OP50 control strain. We targeted five strains carrying mutations on genes belonging to three different regulatory pathways: the insulin pathway (*daf-2* and *daf-16*), the p38 MAPK pathway (*sek-1* and *skn-1*), and the JNK pathway (*jnk-1*). In the case of mutants of the insulin pathway, we did not observe a significant difference (*p* = 0.7) in the longevity of *daf-2* mutant (CB1370) nematodes (Figure 1A) according to whether they were fed with *E. coli* OP50 or *L. rhamnosus* Lcr35^®^. Conversely, for the *daf-16* mutant (GR1307; Figure 1B), we note a significant difference (*p* = 0.008). This difference is not visible on median survival or longevity but in the presence of *L. rhamnosus* Lcr35^®^, a larger proportion of the population survived longer. Regarding the mutants of the p38 MAPK pathway, we did not observe any significant difference in the longevity of both *sek-1* mutant (KU4; *p* = 0.9, Figure 1C) and *skn-1* mutant (LG333; *p* = 0.07, Figure 1D) nematodes. However, due to the severe mutation of the *skn-1* gene (partially rescued) in the LG333 mutant, caution should be exercised in interpreting the results. Indeed, although it looks like that the mutation has no effect, the use of a balanced heterozygous mutant would be necessary to clarify the involvement of *skn-1* gene. On the other hand, we have demonstrated a significant increase in the longevity (*p* < 2.10^−16^) of the *jnk-1* mutant (VC8) of 14.3% and an increase of 30% of the median survival, in the presence of *L. rhamnosus* Lcr35^®^ (Figure 1E) which in that case, exhibit a strong prolongevity activity toward the host.

### 3.2. *Caenorhabditis elegans* loss-of-function mutants survival assay in a context of a candidiasis preventive treatment

During this experiment, our goal was to determine the impact of mutations on the DAF-2/DAF-16 and p38 MAPK signaling pathways on *L. rhamnosus* Lcr35^®^ anti-*C. albicans* properties.

**Figure 2:**
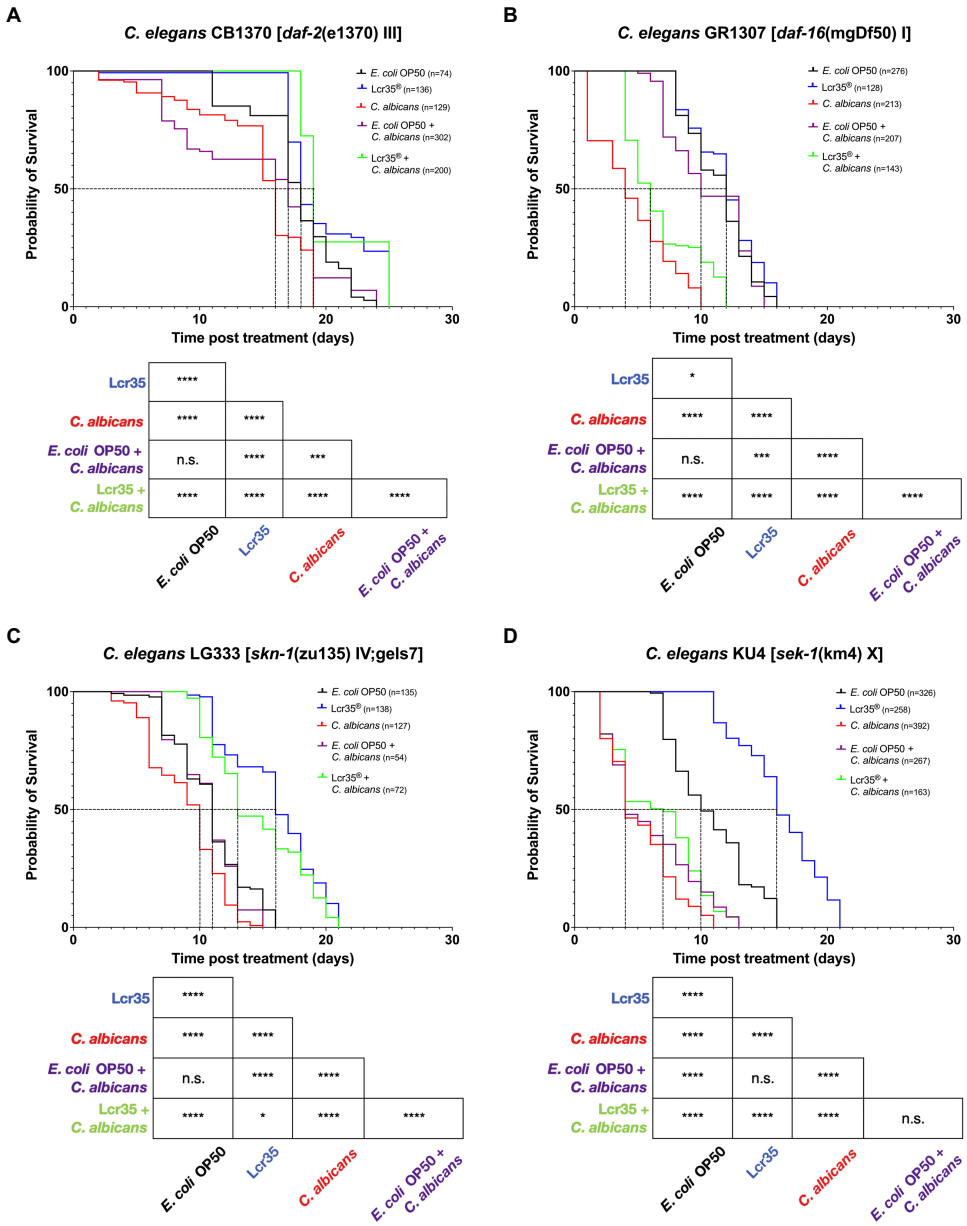
Preventive effects of Lcr35^®^ against *C. albicans* in *C. elegans daf-2* (CB1370) **(A)**, *daf-16* (GR1307) **(B)**, *sek-1* (KU4) **(C)**, and *skn-1* (LG333) **(D)** loss-of-function mutant strains. The mean lifespan, where half of the population was dead, is represented on the abscissa. The asterisks indicate the value of *p*s (log-rank test) with *E. coli* OP50 as a control (NS, not significant; *p < 0.05; ***p < 0.001; ****p < 0.0001).

In the case of a mutation on the *daf-2* gene (CB1370 mutant; Figure 2A), we observed a significant decrease in the survival of *C. elegans* in the presence of *C. albicans* (*p* = 1.10^−7^) with a reduction of 11.1% of median survival compared to the *E. coli* OP50 control condition. The longevity of the host was significantly increased to 25 days in the presence of *L. rhamnosus* Lcr35^®^ (*p* = 8.10^−6^). In addition, mortality in the population was observed only after 17 days. For preventive treatment with *E. coli* OP50, we did not observe any significant decrease in the overall survival of the nematode (*p* = 0.1) although we observed relatively early mortality in the population. Finally, preventive treatment with *L. rhamnosus* Lcr35^®^ induced a significant increase (*p* = 3.10^−14^) in the mean lifespan and in the longevity of the nematodes despite the infection by the pathogen.

If we consider the mutation of the *daf-16* gene (GR1307 mutant; Figure 2B), it induced an increased susceptibility to fungal infection. Indeed, the presence of *C. albicans* resulted in a rapid mortality of worms with a reduction (*p* < 2.10^−16^) of the median survival and longevity of 66.7 and 37.5%, respectively. As for the presence of the LBM *L. rhamnosus* Lcr35^®^, this allowed only a small increase in the overall survival of the animal (*p* = 0.02). Also, when a preventive treatment with *E. coli* OP50 was applied (i.e., *E. coli* OP50 then *C. albicans*), it did not involve any modification of nematode survival compared to the control condition *E. coli* OP50 (*p* = 0.1). In contrast, *L. rhamnosus* Lcr35^®^ as a preventive treatment resulted in a significant decrease in host survival (*p* < 2.10^−16^) with a 50% reduction in the mean lifespan and a 25% reduction in longevity compared to the *E. coli* OP50 control condition.

With the mutation of the gene *sek-1* (KU4 mutant; Figure 2C), we found that the pathogen *C. albicans* induced a negative effect on the worm survival compared to the *E. coli* OP50 control condition (*p* < 2.10^−16^). Since worms were fed with *E. coli* OP50 prior to be infected by the pathogen, no impact on the worm survival to candidiasis was observed. Compared to the control condition, this translates into a significant decrease in nematode survival (*p* < 2.10^−16^) with a 60% reduction in the mean lifespan. In the same way, it turns out that a preventive treatment with the LBM *L. rhamnosus* Lcr35^®^ does not make it possible to extend the survival of the host against candidiasis (*p* < 2.10^−16^). Moreover, there was no significant difference (*p* = 0.2) in terms of survival, depending on whether the worms received preventive treatment with *E. coli* OP50 or *L. rhamnosus* Lcr35^®^.

Finally, with the mutant *skn-1* (LG333 mutant; Figure 2D), we noted similar trends for worms in the presence of *C. albicans* or *L. rhamnosus* Lcr35^®^ as previously stated. Indeed, *C. albicans* induced a reduction of *C. elegans* survival (*p* = 3.10^−7^), while *L. rhamnosus* Lcr35^®^ induced an increase of survival (*p* < 2.10^−16^) compared to *E. coli* OP50. During the preventive treatment with *E. coli* OP50, we noted that it did not induce any significant difference in the survival of the host (*p* = 0.3). On the other hand, a preventive treatment with *L. rhamnosus* Lcr35^®^ allowed a significant increase in the longevity of the nematode (*p* = 3.10^−12^). As in the previous experiment, the mutation of the *skn-1* gene (partially rescued) requires careful interpretation since a balanced heterozygous mutant could induce a different response.

To sum up, all the results showed the negative impact of yeast and the positive impact of LBM on host survival, regardless of the mutant. Analysis of the survival curves showed that the protective effect of *L. rhamnosus* Lcr35^®^ against candidiasis was not impacted by the mutations of *daf-2* and *skn-1* but was abolished by the mutations of *daf-16* and *sek-1*.

### 3.3. Impact of *Lacticaseibacillus rhamnosus* Lcr35^®^ or *Candida albicans* single strains on *Caenorhabditis elegans* whole transcriptome

**Figure 3:**
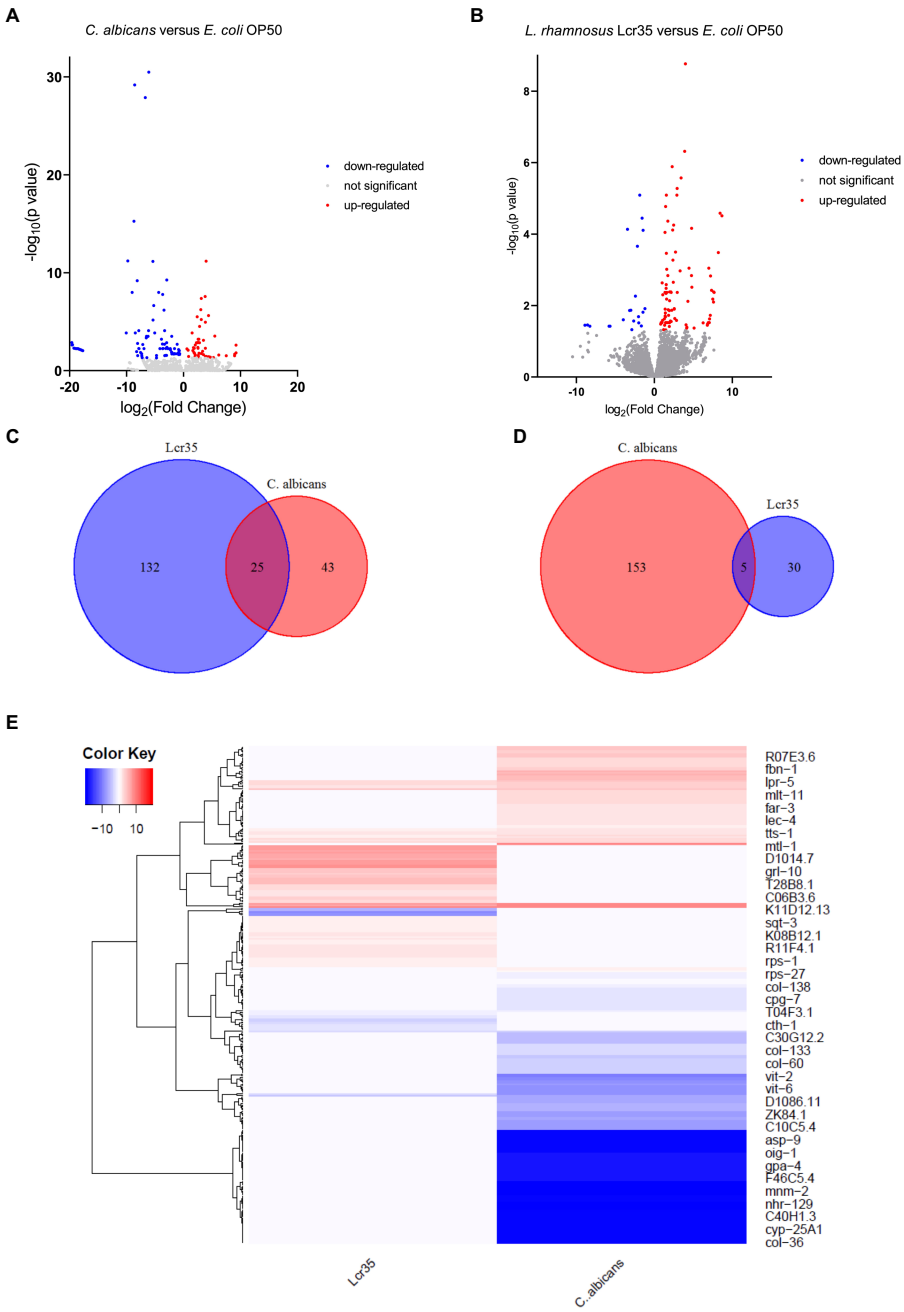
Transcriptomic analysis of *C. elegans* N2 fed with *L. rhamnosus* Lcr35^®^ or *C. albicans* ATCC 10231. **(A)** Volcano plot displaying differentially expressed genes between *C. albicans* and *E. coli* OP50 conditions. **(B)** Volcano plot displaying differentially expressed genes between *L. rhamnosus* Lcr35^®^ and *E. coli* OP50 conditions. The transcriptional responses to *C. albicans* and *L. rhamnosus* Lcr35^®^ comprise specific and overlapping gene sets. Venn diagrams illustrate the overlap genes significantly upregulated **(C)** or downregulated **(D)** by *C. albicans* and *L. rhamnosus* Lcr35^®^. **(E)** Clustering heatmap of differentially expressed genes. Red color indicating significantly highly expressed genes and blue color indicating significantly down-regulated genes. Animals were exposed to the microbial strains for 4 h. The exhaustive list of genes is given in Supplementary Tables S1, S2.

To detect differentially expressed genes between the different experimental conditions, a value of *p* > 0.05 was regarded as a threshold. Volcano plots (Figures 3A,B) show a graphical representation of these differential expressions. They emphasize the variations in expression levels between the experimental conditions but also in absolute value between the under- and over-expressed genes. Indeed, the pathogen *C. albicans* caused overexpression in 43 genes and repression in 153 genes of which 71 present a very strong magnitude of change (between −17 and −20 log_2_ fold change) and three (*col-81*, *col-129*, *col-139*) are the most statistically significant (log_10_ value of *p* of around 30). In contrast, they revealed that *L. rhamnosus* Lcr35^®^ induced the overexpression of 132 genes (in particular *lys-3*, log_2_FC = 7.67 and -log_10_ value of *p* = 2.36) and the repression of 30 genes (*elo-5* and *elo-6* being the most statistically significant). Using a comparative analysis, we determined the gene sets expressed differentially in a specific way or the overlapping gene sets. Venn diagrams (Figures 3C,D) revealed that 25 genes are overexpressed (in particular *col-10*, *col-42*, *col-98*, and *col-103*), and 5 genes are repressed both in the presence of bacteria and yeast. The exhaustive lists of significantly differentially expressed genes are presented in Supplementary Table S1 for upregulated genes and Supplementary Table S2 for downregulated genes. We also conducted a cluster analysis to compare the expression patterns induced by *L. rhamnosus* Lcr35^®^ and *C. albicans*. The heatmap (Figure 3E) obtained indicates a strong dissimilarity in the transcriptional profile induced by the two microorganisms.

**Figure 4:**
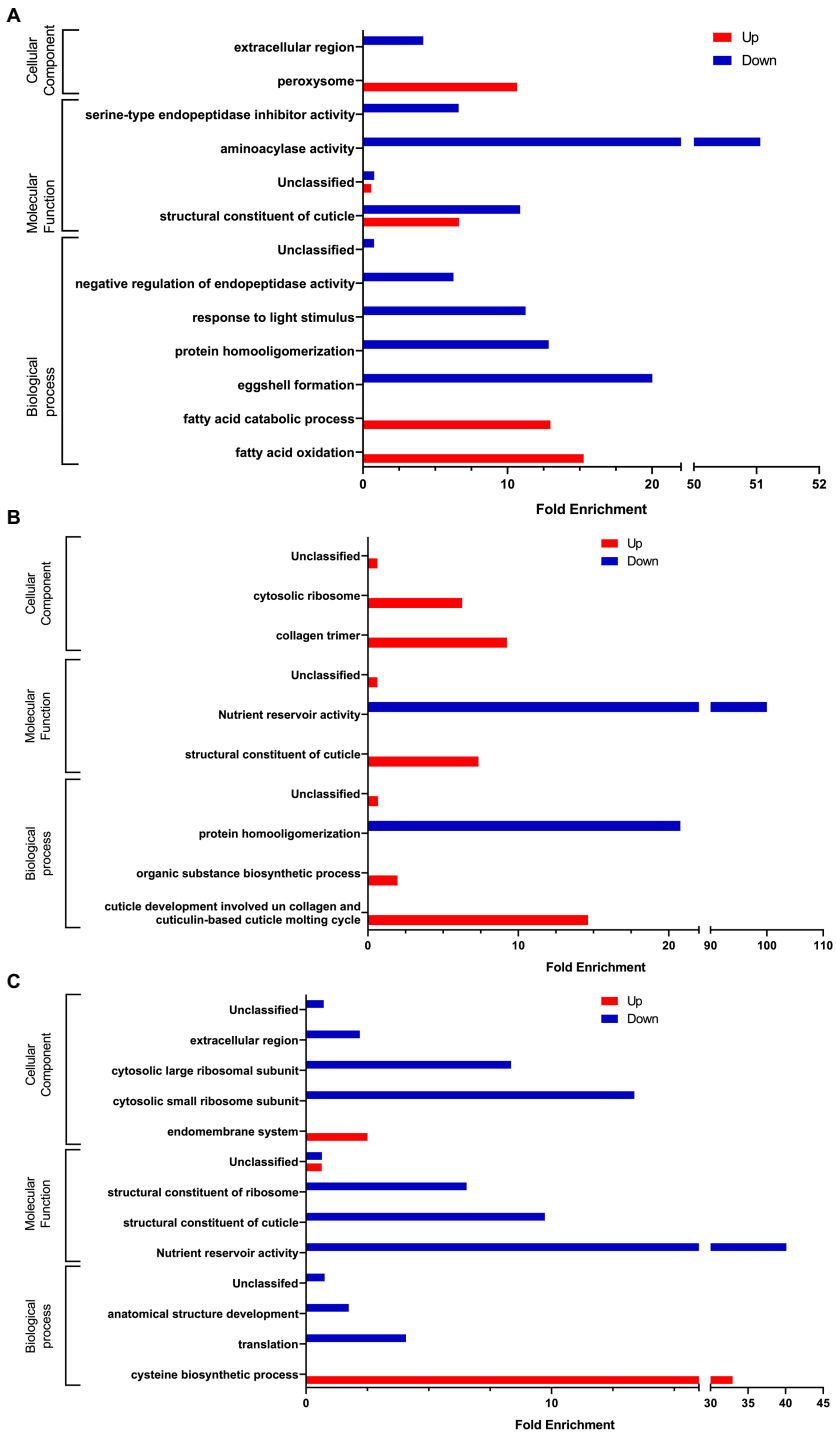
Gene Ontology analysis of differentially expressed genes **(A)** in presence of *C. albicans* versus *E. coli* OP50, **(B)** in presence of *L. rhamnosus* Lcr35^®^ versus *E. coli* OP50, and **(C)** in presence of *C. albicans* versus *L. rhamnosus* Lcr35^®^. Only GO terms significantly enriched (value of *p* and false discovery rate < 0.05) are displayed.

In the presence of *C. albicans* (Figure 4A), we observed a significant enrichment of the biological processes involved in lipid catabolism (fatty acid oxidation and fatty acid catabolic process). This catabolism was pointed out by the cellular component of the peroxisome. On the other hand, if we consider the down-regulated genes we noticed a significant enrichment of terms related to the regulation of endopeptidase activity and protein homooligomerization which is associated with serine-type endopeptidase inhibitor activity and the aminoacylase activity as well. Eggshell formation, associated with structural constituent of cuticle and extracellular region are GO terms significantly enriched as well. Surprisingly, we also noticed a significant enrichment of response to light stimulus.

In the presence of the *L. rhamnosus* Lcr35^®^ strain, the overexpressed genes (Figure 4B) were involved in totally different biological processes and were rather structural in nature. Indeed, we observed a significant enrichment of the term related to cuticle development involved in collagen and cuticulin-based cuticle molting cycle. Similar GO term was also found at the level of molecular functions, with the term structural constituent of cuticle. The collagen trimer is particularly represented. We also noted ontologies related to organic substance biosynthetic process and cytosolic ribosome. As for the under-regulated genes, they are involved in protein homooligomerization. From a molecular point of view, we noted that the genes were also related to nutrient reservoir activity.

We also compared worm transcriptome in the presence of *C. albicans* to that in the presence of *L. rhamnosus* Lcr35^®^. As previously, our results showed that upregulated genes are associated with the activation of metabolic processes ontologies (Figure 4C) notably in connection with the cysteine biosynthetic process but not associated with any molecular function or cellular component. Conversely, regarding the downregulated genes, it is again the structural response that is significantly enriched with terms related to the anatomical structure development, associated with some molecular function and cellular components GO terms. In addition, translation, cytosolic small ribosome subunit and cytosolic large ribosome subunit appeared in the GO terms.

### 3.4. Impact of anti-candidiasis treatment on *Caenorhabditis elegans* whole transcriptome

#### 3.4.1. *Lacticaseibacillus rhamnosus* Lcr35^®^ preventive treatment

**Figure 5:**
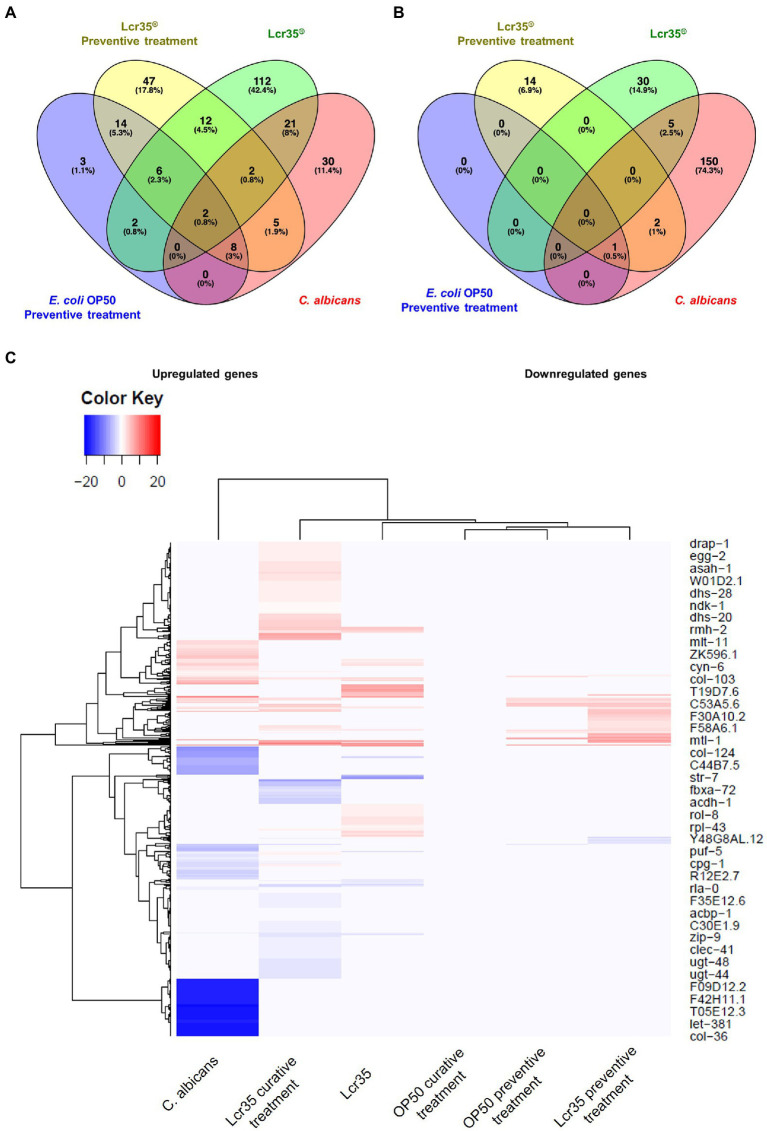
The transcriptional responses to *E. coli* OP50 as a preventive treatment and *L. rhamnosus* Lcr35^®^ as a preventive treatment comprises specific and overlapping gene sets. Venn diagrams illustrate the overlap genes significantly upregulated **(A)** or downregulated **(B)**. Animals were exposed to the bacterial strains for 4 h and infected by the yeast for 2 h. **(C)** Clustering heatmap of differentially expressed genes during a preventive or a curative treatment. Red color indicating significantly highly expressed genes and blue color indicating significantly down-regulated genes. The exhaustive list of genes is given in Supplementary Tables S3, S4.

**Figure 6:**
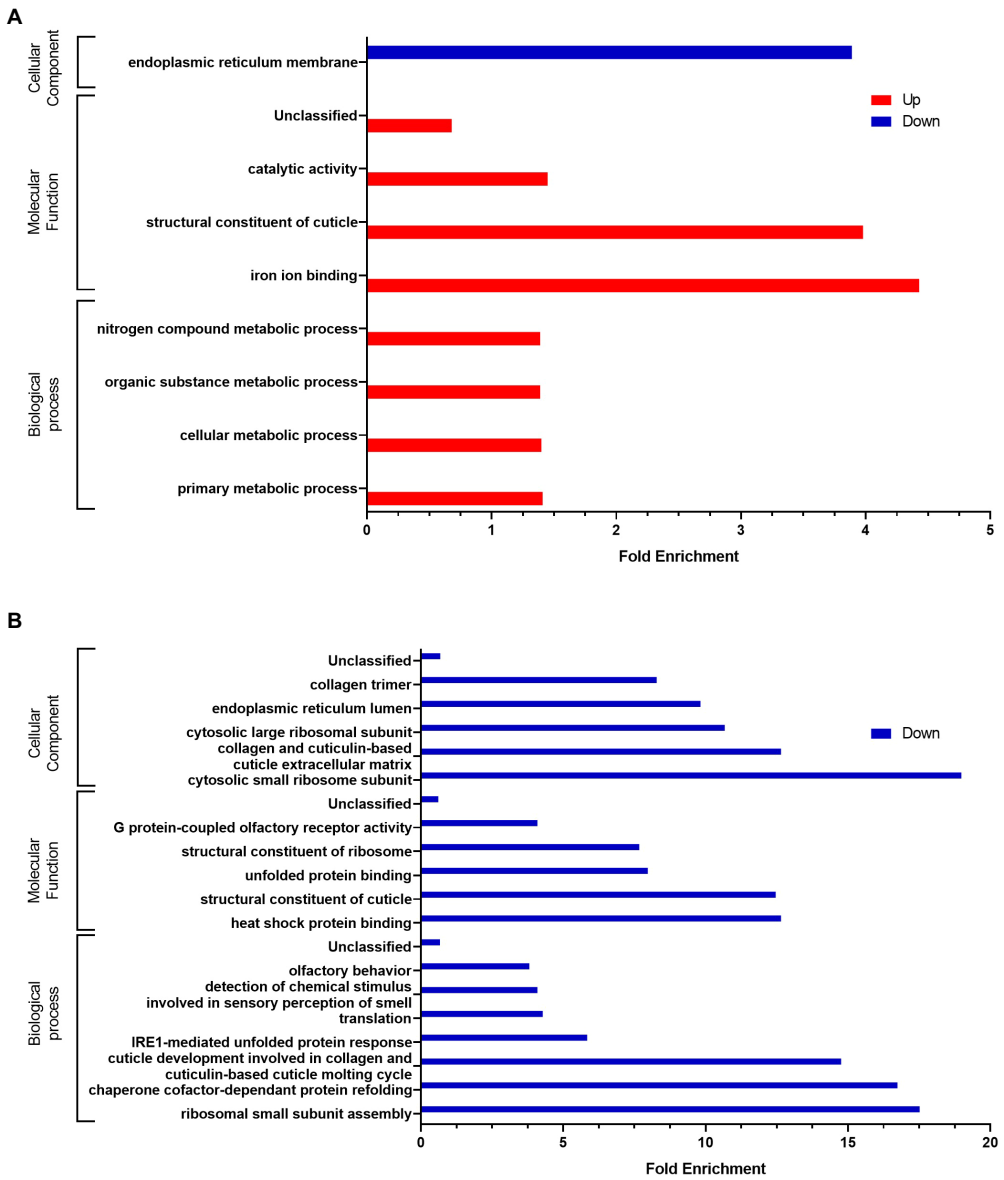
GO analyses of differentially expressed genes after **(A)** a *L. rhamnosus* Lcr35^®^ preventive treatment compared to an un-treated candidiasis or **(B)** compared to *L. rhamnosus* Lcr35^®^. Only GO terms significantly enriched (value of *p* and false discovery rate < 0.05) are displayed.

Sequentially incubating *C. elegans* with *E. coli* OP50 or *L. rhamnosus* Lcr35^®^ and then with *C. albicans* induced modifications to the host transcriptome as compared to incubation on a single strain. Volcano plots (Supplementary Figures S2A,B) show strong differences in terms of expression levels between the different experimental conditions. Using a finer analysis with Venn diagrams (Figures 5A,B), we noted that 72 genes (27.4%) significantly overexpressed are in common in at least two different experimental conditions. In addition, differential analysis showed that most genes were expressed specifically when the worm has been in contact with *L. rhamnosus* Lcr35^®^ (alone or preventively). Interestingly, we noticed that the genes significantly repressed in the nematode, were specific to the condition considered. Indeed, only 3 genes have their expressions significantly repressed both in condition with *C. albicans* alone and during preventive treatment with *L. rhamnosus* Lcr35^®^. The exhaustive list of significantly expressed genes is presented in Supplementary Table S3. As previously, we conducted a cluster analysis to compare the expression patterns induced by a preventive treatment with *E. coli* OP50 or *L. rhamnosus* Lcr35^®^. The heatmap (Figure 5C) clearly indicates the presence of a cluster grouping together the two preventive treatments, departing significantly from the condition where the nematode was in contact only with the pathogenic yeast. Despite the presence of yeast after preventive treatment, the transcriptional profile is closer to that in the presence of *L. rhamnosus* Lcr35^®^ alone. Comparison of the preventive treatment with *L. rhamnosus* Lcr35^®^ and the untreated candidiasis showed that significantly under-regulated genes were involved in only one ontology, the endoplasmic reticulum membrane (Figure 6A). For the overexpressed genes, we noted that they were involved in several biological processes with primary, cellular, organic substance and nitrogen compound metabolic processes. Considering the molecular functions, they are linked to structural constituent of cuticle and catalytic activity as well.

The same GO analysis was conducted by comparing the *L. rhamnosus* Lcr35^®^ as a preventive treatment and Lcr35^®^ alone conditions (Figure 6B). The analysis did not reveal any GO terms significantly enriched for the upregulated genes. On the contrary, down-regulated genes were found implied in a variety of ontologies such as the development of cuticle, the translational activity or the detection of molecules to quote only those biological processes. GO terms linked to the structure of the cuticle were also found as molecular functions and cellular component whereas only cellular components comprise terms associated with the translational activity.

#### 3.4.2. *Lacticaseibacillus rhamnosus* Lcr35^®^ curative treatment

**Figure 7:**
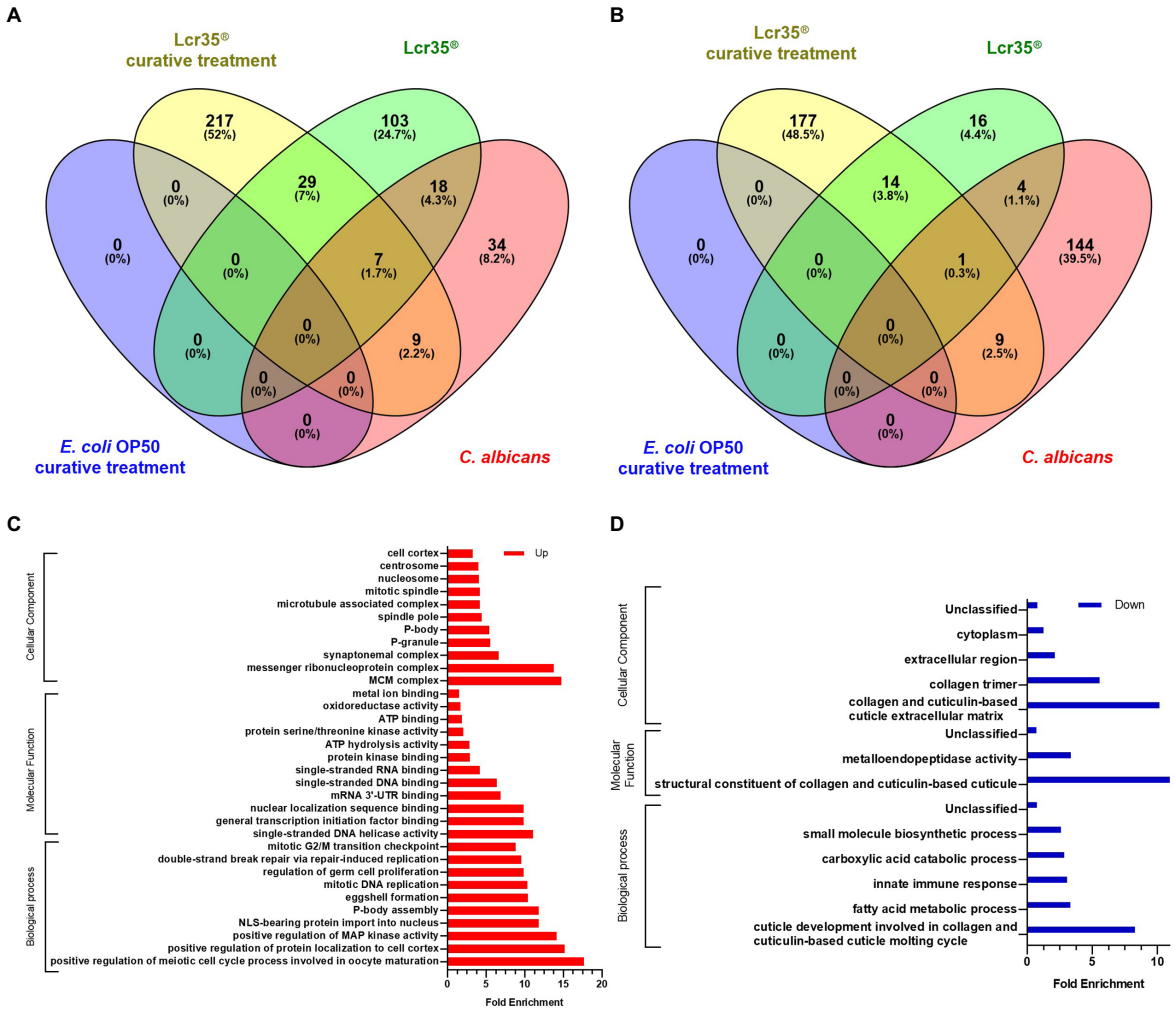
The transcriptional responses to *E. coli* as a curative treatment and *L. rhamnosus* Lcr35^®^ as a curative treatment comprises specific and overlapping gene sets. Venn diagrams illustrate the overlap genes significantly upregulated **(A)** or downregulated **(B)**. Animals were exposed to the bacterial strains for 4 h and infected by the yeast for 2 h. GO analyses of upregulated expressed genes **(C)** or downregulated expressed genes **(D)** after a *L. rhamnosus* Lcr35^®^ curative treatment compared to worms fed with *L. rhamnosus* Lcr35^®^ only. Only GO terms significantly enriched (value of *p* and false discovery rate < 0.05) are displayed.

In the same way as for the preventive treatment of candidiasis, the overall transcriptomic analysis following a curative treatment reveals the presence of genes expressed in a specific way or overlapping depending on the experimental conditions (Figures 7A,B and Supplementary Figure S2D). In the present case, we noted that the differential expression is mainly specific with 217, 103, and 34 overexpressed genes for the conditions Lcr35^®^ curative treatment, Lcr35^®^ and *C. albicans*, respectively. As for the overlapping genes, these represent only 10.9% of the total, and are expressed mainly in the Lcr35^®^ and Lcr35^®^ curative treatment conditions. Among them, we found *hrg-3*, *hrg-4* and *hrg-7* involved in heme binding and transmembrane transport, *rps-7*, *rps*-*11* and *rps*-*14*, predicted to be a structural constituent of ribosome, or *col*-103, predicted to be a structural constituent of cuticle. For the under-expressed genes, a similar trend is present with 117 and 144 specific genes for Lcr35^®^ curative treatment (*C. albicans* then *L. rhamnosus* Lcr35^®^) and *C. albicans* conditions, respectively. Overlapping genes represent 6.6% of the total and include *elo-5*, *elo-6*, predicted to enable fatty acid elongase activity, *nhr-114*, a nuclear hormone receptor, or *col-71*, *col-133* and *col-138*, predicted to be structural constituents of cuticle. More interestingly, we noted that no gene is significantly differentially expressed when the nematode received a curative treatment with *E. coli* OP50, in comparison with the control condition (*E. coli* OP50 only; Supplementary Figure S2C).

Presented in Figures 7C,D, in comparison with the transcriptome of nematodes fed exclusively with *L. rhamnosus* Lcr35^®^, that of nematodes having received a curative treatment against candidiasis with Lcr35^®^ presents different significantly enriched GO terms depending on whether the genes are over (Figure 7C) or under expressed (Figure 7D). In the first case, we noted a certain diversity in the biological processes implemented with the meiotic and mitotic cell cycles, the replication and the repair of the DNA or the regulation of the activity of the MAP kinases. In contrast, for molecular functions, we noted two main categories targeting metabolism (redox, ATP binding and hydrolysis, kinase activity) and nucleic acids (DNA and RNA binding, helicase activity, transcription). Conversely, under-expressed genes are involved in GO terms involved in the host defense with the innate immune response, in the metabolic activity with the metabolism of fatty acids, carboxylic acids and small metabolites and clearly of a structural nature with the development of the cuticle and molting cycles. These latter biological processes GO terms are to be associated to the molecular functions of the structure of collagen and the cuticle, but also to the cellular components related to collagen, extracellular region and cytoplasm. It is interesting to note a significant enrichment of the molecular function involved in the metalloendopeptidase activity.

**Figure 8:**
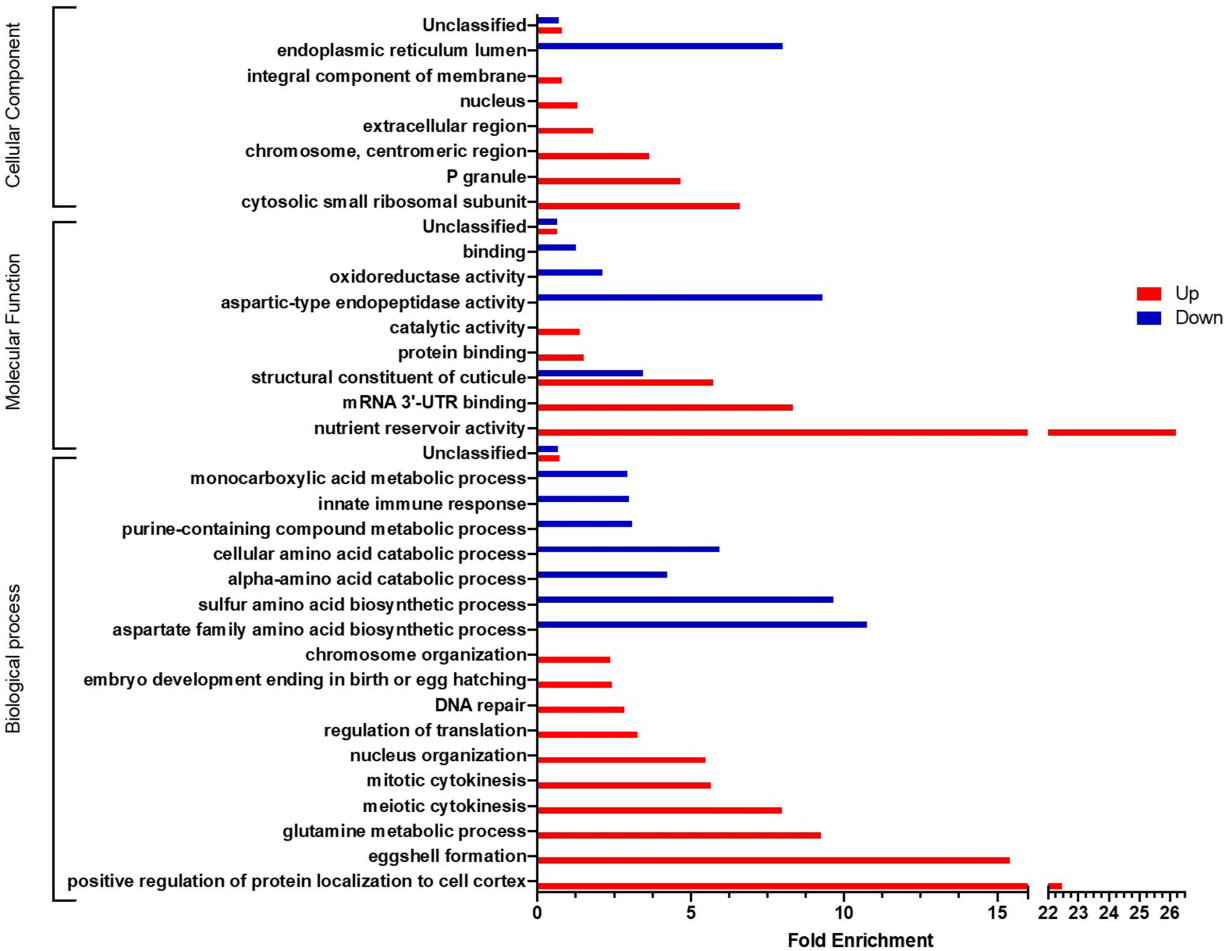
GO analyses of differentially expressed genes after a *L. rhamnosus* Lcr35^®^ curative treatment compared to an un-treated candidiasis. Only GO terms significantly enriched (value of *p* and false discovery rate < 0.05) are displayed.

Similarly, a comparison of the transcriptome of nematodes that received a curative treatment with *L. rhamnosus* Lcr35^®^ with that of nematodes infected with *C. albicans*, we observed a notable dichotomy depending on whether the GO terms are associated with over- or under-expressed genes (Figure 8). Indeed, if we consider biological processes, overexpressed genes are involved in processes related to reproduction (eggshell formation, embryo developing ending in birth or egg hatching), cell multiplication (meiotic cytokinesis, mitotic cytokinesis) and DNA (nucleus organization, regulation of translation, DNA repair, chromosome organization). Conversely, most under-expressed genes are more involved in metabolic processes, in particular amino acid metabolism or the immune response. Concerning the molecular functions, the structural term constituent of cuticle is both over- and under-enriched, which is also the case for terms associated with metabolic functions. On the other hand, for the cellular components, we found a distribution similar to biological processes, namely, terms related to nucleic acids and the nucleus are over-enriched while the endoplasmic reticulum, seat of peptide synthesis, is under-enriched.

From a global point of view, the analysis of the expression profile *via* a clustering represented by the heatmap (Figure 5C) makes possible to highlight the proximity level of the Lcr35^®^, Lcr35^®^ curative treatment and Lcr35^®^ preventive treatment conditions as well as their distance from the *C. albicans* condition. Indeed, it induces a very different alteration of the transcriptome of *C. elegans* in terms of number of differentially expressed genes (downregulated genes: 201 genes during a curative treatment versus 17 genes during a preventive treatment; upregulated genes: 262 genes during a curative treatment versus 97 genes during a preventive treatment). The exhaustive list of significantly expressed genes is presented in Supplementary Table S4.

#### 3.4.3. Antioxidant assay

**Figure 9:**
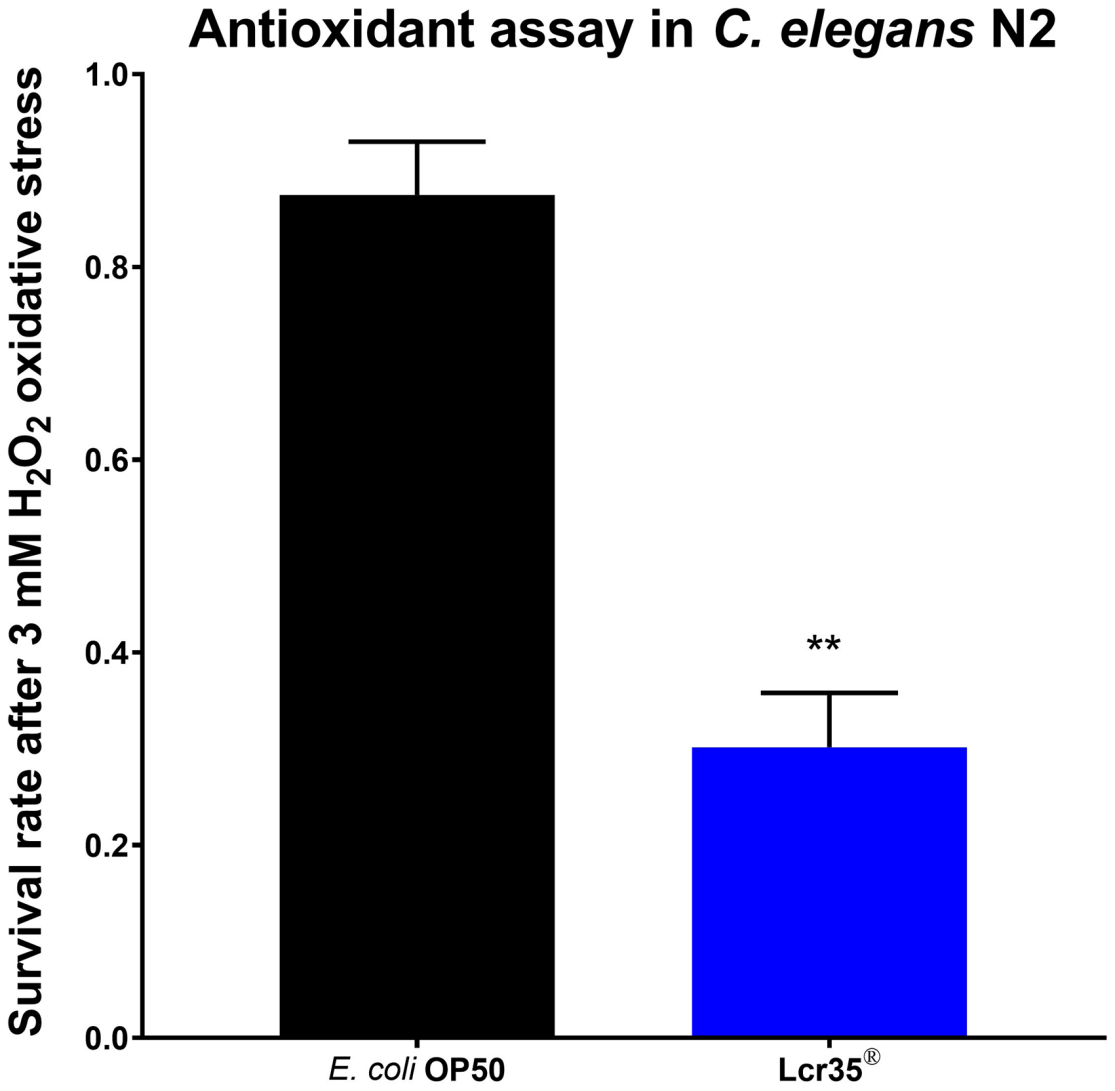
Antioxidant assay in *C. elegans*. Survival upon a 3 mM H_2_O_2_ treatment for 3.5 h of *C. elegans* N2 strain fed for 5 days with *E. coli* OP50 or Lcr35^®^ (**p < 0.01).

In order to examine the antioxidant properties of *L. rhamnosus* Lcr35^®^, we fed the worms (wild-type) with the probiotic and we tested *C. elegans* survival rate σ_r_ upon H_2_O_2_-induced stress. This was performed by measuring worm viability after 3.5 h on medium supplemented with hydrogen peroxide. We observed a significant decrease (*p* = 0.002) in the survival rate of nematodes according to whether they were previously fed with the control strain *E. coli* OP50 (87.5 ± 5.5%) or the probiotic *L. rhamnosus* Lcr35^®^ (30.2 ± 5.7%), with a loss of viability of 65.5% (Figure 9).

## 4. Discussion

Numerous studies have demonstrated the interest of *C. elegans* implementation in order to screen collections of microorganisms and to identify potential candidates with probiotic properties (Lee et al., 2015; de Barros et al., 2017; Chelliah et al., 2018). For strains with a proven probiotic character, *C. elegans* is also a relevant model for the conduct of mechanistic studies (Park et al., 2018; Poupet et al., 2019a,b,c) due to the availability of a large collection of mutants and, because of its genetic and protein homologies with humans (Zhang et al., 2017). Likewise, the nematode constitutes a tool allowing the investigation of the pathogenicity of human microorganisms, whether they are bacterial (Hwang et al., 2018; King et al., 2018) or fungal (Pukkila-Worley et al., 2009, 2011; Peterson et al., 2019).

Although some authors have been able to demonstrate the involvement of certain regulatory pathways during the microbial strain probiotic effect, very few studies have been conducted in order to comprehensively decipher these mechanisms by high-throughput methods (DNA micro-array, NGS). In their review, Dierking et al. (2016) pointed out that non-pathogenic bacteria, like *L. rhamnosus*, had no overall impact on the expression of genes coding for molecules with antimicrobial activity. Only the *spp-6* (up-regulation), *spp-16*, *spp-23*, *lys-1* and *lys-6* (down regulation) genes have their expressions modulated (Dierking et al., 2016). In our case, the overall transcriptomic analysis shows that Lcr35^®^ has relatively little impact on the transcriptome of *C. elegans*. Considering the defense genes, only *lys-3* (encoding for a lysozyme), and *lpr-3*, *lpr-5* and *lpr-6* (encoding for lipocalin-related proteins and predicted to be involved in the immune response) showed a significant overexpression. Also, we noted the overexpression of genes involved in the structural response and the lipid metabolism (*bli-2*, *col-10*, *cuti-1*, *acs-7*, *fat-3*). More specifically, a study focused on the impact of the *L. rhamnosus* CNCM I-3690 strain on the transcriptome of *C. elegans*. The authors highlighted that some overexpressed genes (*lec-11*, *spp-16*, *clc-1*, *gst-22*, *fat-7*, *acl-12*, *hsp-12.3*) in young worms were involved in the insulin-like pathway, in lipid metabolism as well as in the stress response (Grompone et al., 2012) which is not the case in our study. The comparison of all these data shows that the modulation of the transcriptome of *C. elegans* in the presence of a probiotic seems to be strain-specific so that an inter-species or even inter-genus generalization seems risky.

Unlike the properties of probiotics, the pathogenesis of microorganisms, in particular *C. albicans*, is rather well described in the literature (Pukkila-Worley et al., 2009, 2011). By focusing on the modulation of the nematode transcriptome induced by *C. albicans*, we realized that there was a significant repression of the genes involved in the synthesis of the cuticle but, surprisingly, we noted a marginal modulation of genes encoding antifungal peptides. In effect, contrary to what we had shown in our previous works (Poupet et al., 2019a,b,c), we were not able to show a significant differential expression of these genes (*abf-2*, *cnc-4*, *cnc-7*, *fipr-22*, and *fipr-23*) in the present study. On the other hand, high-throughput RNA sequencing revealed an overexpression of the *nlp-29* gene, an antimicrobial peptide also dependent on PMK-1, involved in antifungal defense (Pujol et al., 2008). This absence of modulation of the immune response is somewhat unexpected since it goes against previous published data. According to the work of Pukkila-Worley and colleagues, after a fungal infection, a rapid transcriptional response takes place in the nematode *via* the modulation of 313 genes, approximately 1.6% of the genome. Most of these genes encode for antimicrobial molecules as well as secreted or detoxifying proteins. Among these genes, the authors have shown the overexpression of antimicrobial genes such as *abf-2*, *cnc-4*, *cnc-7*, *fipr-22*, and *fipr-23*. Except for *abf-2*, all these genes are under the control of PMK-1, whose inactivation makes the nematode susceptible to the infection (Pukkila-Worley et al., 2011). This different transcriptional response can probably be explained by the fact that the studies involve two distinct strains of *C. albicans* (DAY185 and ATCC 10231) with their own pathogenicities. Likewise, the authors demonstrated the high degree of specificity of the nematode’s immune response. Indeed, only a few genes overexpressed in the presence of *C. albicans* are also expressed with the bacteria *Pseudomonas aeruginosa* and *Staphylococcus aureus.* Also, they have shown that most genes induced by bacteria are repressed in the presence of yeast (Pukkila-Worley et al., 2011). More recently, Sun et al. (2016) revealed that during *C. albicans* infection, dysregulation of microRNA expression (miRNAs) occurred. The authors suggested that three miRNAs, *mir-251*, *mir-252*, and *mir-360*, play an essential role in controlling the regulation of nematode immunity. A loss of function of the first two genes induces an increased resistance to infection while for the last, a susceptibility was observed. Also, the authors indicate that *mir-251* and *mir-252* function downstream of the p38 MAPK pathway or insulin-like (Sun et al., 2016). In addition, these two genes are homologous of the human miRNA *mir-26* (Ibáñez-Ventoso et al., 2008).

So far, many lactic acid bacteria with probiotic properties have been studied in the *C. elegans* worm with the major result of extending the longevity of the host. However, as noted in the review by Roselli and colleagues (Roselli et al., 2019), few studies have allowed the characterization of the molecular mechanisms involved. Otherwise, probiotics effects involve the insulin-like pathway (DAF-2/DAF-16; Ikeda et al., 2007; Grompone et al., 2012; Poupet et al., 2019a,b,c) or the canonical p38 MAPK signaling pathway (Kim and Mylonakis, 2012; Nakagawa et al., 2016; Poupet et al., 2019a,b). In *C. elegans*, DAF-16 is closely related to mammalian FOXO3a, a transcription factor involved in the inflammatory process (Singh and Aballay, 2009) and leading to the modulation of the worm’s health condition and consequently to its lifetime (Sun et al., 2016). The p38 MAPK (also called PMK-1) pathway is activated through phosphorylation by upstream MAPKK (SEK-1), which is activated by MAPKKK (NSY-1). It has a role in regulating life expectancy by modulating the specific immune response of the worm and has been shown to be required in its resistance to bacterial infection (Xu et al., 2013).

In our previous paper, we demonstrated that *L. rhamnosus* Lcr35^®^ induced to *C. elegans* both an increase of longevity and an increase of survival to candidiasis. Here, we implemented experiences on longevity and survival against the fungal infection with loss-of-function mutants which allowed us to highlight the implication of different target genes in presence of *L. rhamnosus* Lcr35^®^. In the case of the longevity and compared to the *E. coli* OP50 control condition, a mutation leading to a loss of function of *jnk-1* did not lead to a reduction in the longevity of nematodes in the presence of *L. rhamnosus* Lcr35^®^, thus refuting the involvement of the JNK pathway in the prolongevity properties of the LBM. Conversely, we demonstrated the *daf-2*, *daf-16*, *sek-1* and *skn-1* genes were involved in the *L. rhamnosus* Lcr35^®^ prolongevity mechanism. This is explained by the absence of a significant increase in the longevity of *C. elegans* in presence of probiotic. In the case of the survival analysis, caution is still required for *daf-16* since we observed a delay in the onset of mortality but not an impact of lifespan. In addition, the loss of function in *daf-16* and *sek-1* mutants strongly impacted the anti-*C. albicans* protective properties of *L. rhamnosus* Lcr35^®^. In these cases, in addition to having a high susceptibility to fungal infection, the presence of *L. rhamnosus* Lcr35^®^ did not provide effective protection of the host. Conversely, the antifungal abilities of *L. rhamnosus* Lcr35^®^ were conserved in the *daf-2* and *skn-1* mutants, which suggests that these two genes play no role in the anti- *C. albicans* effects. Also, we noticed that these two mutants showed a notable resistance to the pathogenic yeast. These results support our previous study where we showed by quantitative RT-PCR that *L. rhamnosus* Lcr35^®^ stimulated the host response through the insulin-like and p38 MAPK pathways (Poupet et al., 2019a,b). Current work has enabled us to demonstrate that *daf-16* and *sek-1* functionalities are required for *L. rhamnosus* Lcr35^®^ to exert its anti-*C. albicans* activity, in knockout mutants for these genes, the protective effect being abolished. Recently, Zhou et al. (2021) investigated the anti-*Salmonella* Typhimurium DT104 capacities of *Lactobacillus zeae* LB1 using a preventive approach. They thus highlighted the overexpression of genes such as *daf-16* (insulin pathway), *tir-1*, *sek-1* and *pmk-1* (p38 MAPK pathway) and *clec-60*, *sod-3* and *skn-1* (other defense molecules) in the presence of the lactobacillus. Furthermore, loss-of-function mutants for the *nsy-1*, *sek-1*, *pmk-1*, *abf-3* and *lys-7* genes are insensitive to the antipathogenic effect of *L. zeae*. According to the authors, these results suggest the bacterium can regulate the host cell signaling *via* p38 MAPK pathway (Zhou et al., 2021). A similar conclusion was also advanced by the work of Dinić et al. (2021) which showed that administration of the heat-inactivated probiotic strain *Lactobacillus curvatus* BGMK2-41 induced a PMK-1/p38 MAPK-dependent transcription of C-type lectins (*clec-85*, *clec-172*), lysozymes (*lys-1*, *lys-3*, *lys-5*, *lys-8*) and tight junction protein CLC-1. This induction has the effect of protection against Gram-positive (*Staphylococcus aureus*) and Gram-negative (*Pseudomonas aeruginosa*) pathogens (Dinić et al., 2021). Another recent study also highlighted the central role of the PMK-1/p38 MAPK pathway in the antipathogenic response induced by *Lacticaseibacillus rhamnosus* GG. However, the authors found that the modulation of the immune response directed against foodborne pathogens (*Salmonella* Typhimurium SL1344, *Staphylococcus aureus* RN6390 and *Enterococcus faecalis* MMH594), and *a fortiori* longevity, took place through the regulation of the microRNA *miR-34*. In the absence of *miR-34*, the authors note the absence of modulation of the expression of PMK-1 during stimulation by LGG (Yun et al., 2022).

More marginally, some authors have highlighted the involvement of the c-Jun N-terminal kinase (JNK) pathway in the presence of probiotics not belonging to the genus *Lactobacillus* (Zhao et al., 2017). The JNK (c-Jun N-terminal Kinase) pathway is activated by cytokines and in response to stresses such as UV irradiation, Reactive Oxygen Species (ROS), DNA damage, heat stress and inflammation (Wolf et al., 2008). In *C. elegans*, this pathway evolves in parallel with the insulin signaling pathway till it converges to DAF-16 which is phosphorylated by JNK-1 (Marudhupandiyan and Balamurugan, 2017). Beyond the extension of longevity, probiotics also exert an antipathogenic activity *via* mainly the three previously mentioned pathways but also *via* the TGF-β, β-catenin and AMPK pathways (Lee et al., 2015; Sim et al., 2018; Yuen and Ausubel, 2018).

Aerobic metabolism inexorably leads to the production of oxidative metabolites, such as reactive oxygen species, which are harmful to cells. An imbalance between these metabolites and detoxification mechanisms (for example superoxide dismutase or catalase) induces cellular damages which can lead to cell death. However, in low concentrations, they have been shown to be beneficial and promote increased longevity. This is particularly the case in *C. elegans* (Schifano et al., 2019). Lactic acid bacteria have a very active oxidative metabolism and must have detoxification enzymes in order to protect themselves from the deleterious effects of oxidizing species. During our work, we showed that *L. rhamnosus* Lcr35^®^ does not allow the worm to resist hydrogen despite the fact that it induces the activation of DAF-16 (Poupet et al., 2019a,b,c). Therefore, it turns out that the genes under the control of DAF-16 are not regulated solely by this transcription factor but that a more complex control mechanism exists and must be described. Here, the study of differentially expressed genes revealed the significant overexpression of *sod-4*, a gene coding for superoxide dismutase, an oxidoreductase involved in oxidative stress. This can be linked to the cellular component GO term of peroxisome. However, it is important to note that peroxisome is not only involved in the reduction of reactive oxygen species but also the fatty acid metabolism. Also, we did not detect any transcriptional modification for genes coding for catalase or peroxidase, thus constituting a hypothesis of the absence of resistance to hydrogen peroxide. Grompone et al. (2012) have screened a collection of lactobacilli and have shown that only a few strains protect *C. elegans* from oxidative stress, including the strain *L. rhamnosus* CNCM I-3690. Consumption of this bacterium by the worm activates genes downstream of the DAF-16/FOXO pathway (Grompone et al., 2012). A recent study has shown that the bacterium *L. fermentum* promotes the longevity of the worm in a dependent manner with PEPT-1, a peptide involved in the regulation of lipid content, while resistance to oxidative stress is independent of it (Schifano et al., 2019).

The results presented here as part of the preventive treatment of candidiasis with *L. rhamnosus* Lcr35^®^ constitutes an important technical and scientific advance. Indeed, no data concerning such an approach is available in the literature. Our method allows the creation of the first data set obtained by high-throughput transcriptomic analysis of the interaction between three organisms: a probiotic, a pathogen and a host. These new insights will heed to new studies to allow understanding of the mechanisms with the aim of significantly improving the effectiveness of anti-infective drug treatments. In addition, this new knowledge also opens the way to new targets for the screening of strains of therapeutic interest.

## 5. Conclusion

In conclusion, we showed the specific transcriptional response of the nematode, which is dependent on the microorganism encountered, either the LBM Lcr35^®^ or the pathogen or both sequentially in a preventive or curative approach of fungal infection. Overall, it turns out that *C. albicans* tended to induce a metabolic response (catabolism and anabolism of small metabolites) while *L. rhamnosus* Lcr35^®^ rather induced a structural response (cuticle synthesis), which is consistent with the hypothesis of a strengthening of the physical defenses of the host by the probiotic. Furthermore, our results demonstrated that *L. rhamnosus* Lcr35^®^ required functional genes (*daf-16* and *sek-1*) from highly conserved signaling pathways to exert prolongevity and anti *C. albicans* effects to the roundworm *C. elegans*. We found that *L. rhamnosus* Lcr35^®^ significantly stimulates *C. elegans* longevity through DAF-2/ DAF-16 and p38 MAP kinase signaling pathways but not through JNK/DAF-16 signaling pathway nor antioxidant effect. Moreover, this interpretation is strengthened by the finding that the first two signaling pathways are also implied in the anti-candidiasis effects which supports the results obtained during our previous work. Hence, next studies are needed to evaluate the impact precisely of *L. rhamnosus* Lcr35^®^ (alone or as a preventive treatment) on the proteome and metabolome of *C. elegans* in order to obtain a more global and integrative vision of the mechanisms of action of LBM *L. rhamnosus* Lcr35^®^, ranging from the expression of a gene of interest up to the synthesis of the metabolite through their regulations. Finally, these insights could be transposed to the in-depth study of the action mechanisms of other *Lacticaseibacillus* spp. against *Candida* spp. or other pathogens through the establishment of new targets for the screening of strains of interest.

## Data availability statement

The datasets presented in this study can be found in online repositories. The names of the repository/repositories and accession number(s) can be found in the article/Supplementary material.

## Author contributions

CP, ÉR, ST, and GC designed the protocols and scientific approaches. CP, ÉR, ST, GC, ÉG, and SR carried out the experiments. MB, PV, CC, and SB provided the critical feedback. CP wrote the manuscript. AN and SB supervised the project. All authors have read and agreed to the published version of the manuscript.

## Funding

This work was supported by the European funds FEDER and the Auvergne-Rhône-Alpes Region in the form of grants to CP as well as biose^®^ as part of the doctoral thesis of CP. The funder biose^®^ provided support in the form of salaries for AN. Some strains were provided by the CGC, which is funded by NIH Office of Research Infrastructure Programs (P40 OD010440). MGX acknowledges financial support from France Génomique National infrastructure, funded as part of Investissement d’avenir program managed by Agence Nationale pour la Recherche (contract ANR-10-INBS-09).

## Conflict of interest

The authors declare that the research was conducted in the absence of any commercial or financial relationships that could be construed as a potential conflict of interest.

## Publisher’s note

All claims expressed in this article are solely those of the authors and do not necessarily represent those of their affiliated organizations, or those of the publisher, the editors and the reviewers. Any product that may be evaluated in this article, or claim that may be made by its manufacturer, is not guaranteed or endorsed by the publisher.

## Footnotes

^1^ https://www.ncbi.nlm.nih.gov/geo/query/acc.cgi?acc=GSE202877
