# Supplementary material for: In vivo investigation of Lcr35® anti-candidiasis properties in Caenorhabditis elegans reveals the involvement of highly conserved immune pathways
Source: Front Microbiol. 2022 Dec 23;13:1062113. doi: 10.3389/fmicb.2022.1062113 (PMC9816150; doi:10.3389/fmicb.2022.1062113)
Supplement: Supplementary file 5 [file Data_Sheet_1.docx]

Supplementary Material

**Table S1: *C. elegans* upregulated genes in presence of Lcr35^®^, *C. albicans* or both. *C. elegans* in presence of *E. coli* OP50 was used as the control condition. a)** Upregulated genes in presence of Lcr35^®^ ; **b)** Upregulated genes in presence of *C. albicans ;* **c)** Upregulated genes in common. See Excel file Table S1.xslx.

**Table S2: *C. elegans* downregulated genes in presence of Lcr35^®^, *C. albicans* or both. *C. elegans* in presence of *E. coli* OP50 was used as the control condition. a)** Downregulated genes in presence of Lcr35^®^; **b)** Downregulated genes in presence of *C. albicans* ; **c)** Downregulated genes in common. See Excel file Table S2.xslx.

**Table S3** ***C. elegans* differentially expressed genes during a candidiasis preventive treatment.** **a)** Downregulated genes in presence of *E. coli* OP50 as a preventive treatment ; **b)** Downregulated genes in presence of Lcr35^®^ as a preventive treatment ; **c)** Downregulated genes in common ; **d)** Upregulated genes in presence of *E. coli* OP50 as a preventive treatment ; **e)** Upregulated genes in presence of Lcr35^®^ as a preventive treatment **f)** Upregulated genes in common. See Excel file Table S3.xslx.

**Table S4 *C. elegans* differentially expressed genes during a candidiasis curative treatment.** **a)** Downregulated genes in presence of *E. coli* OP50 as a curative treatment ; **b)** Downregulated genes in presence of Lcr35^®^ as a curative treatment ; **c)** Downregulated genes in common ; **d)** Upregulated genes in presence of *E. coli* OP50 as a curative treatment ; **e)** Upregulated genes in presence of Lcr35^®^ as a curative treatment ; **f)** Upregulated genes in common. See Excel file Table S4.xslx.


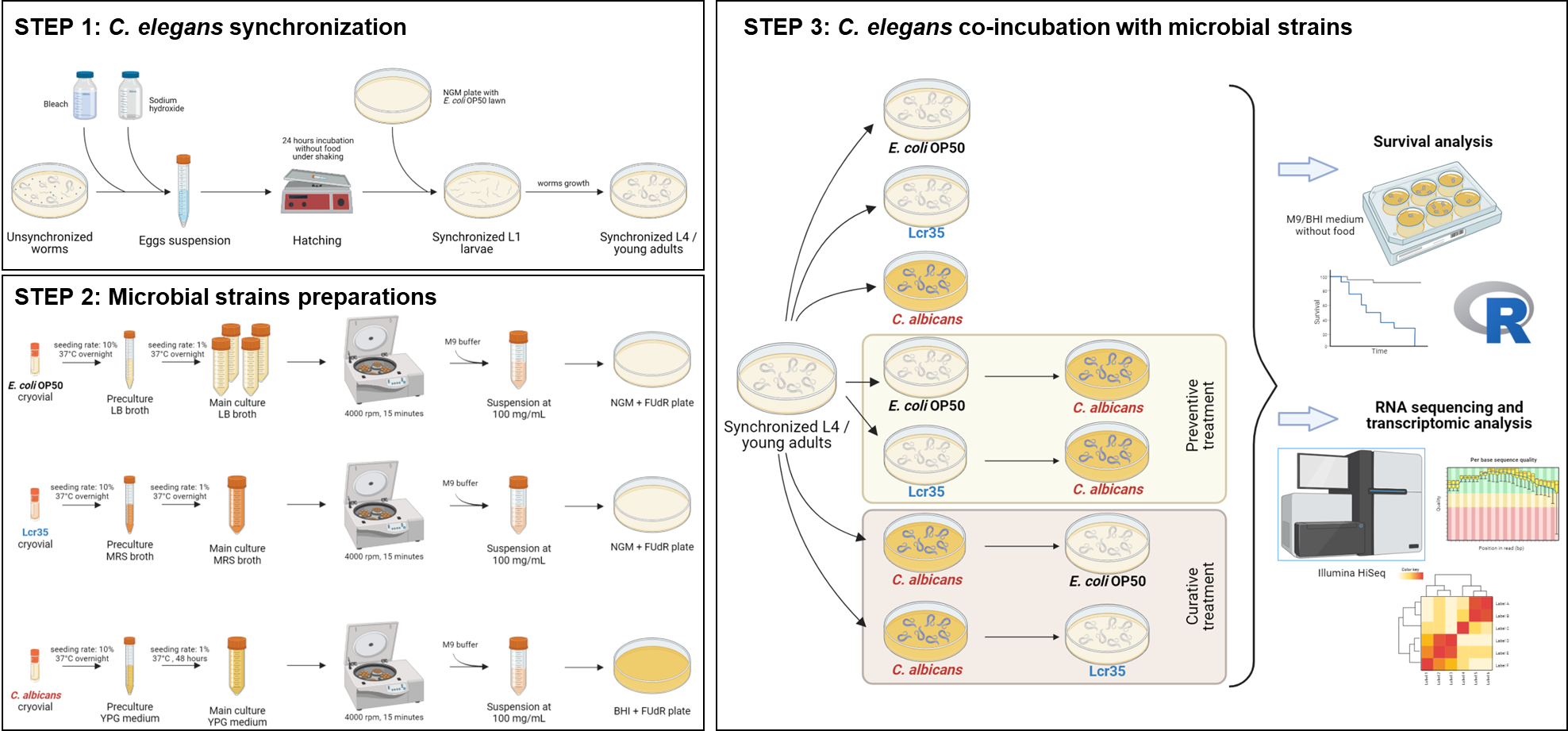


**Figure S1**: Workflow analysis of the effect of bacterial and fungal strains on *C. elegans*


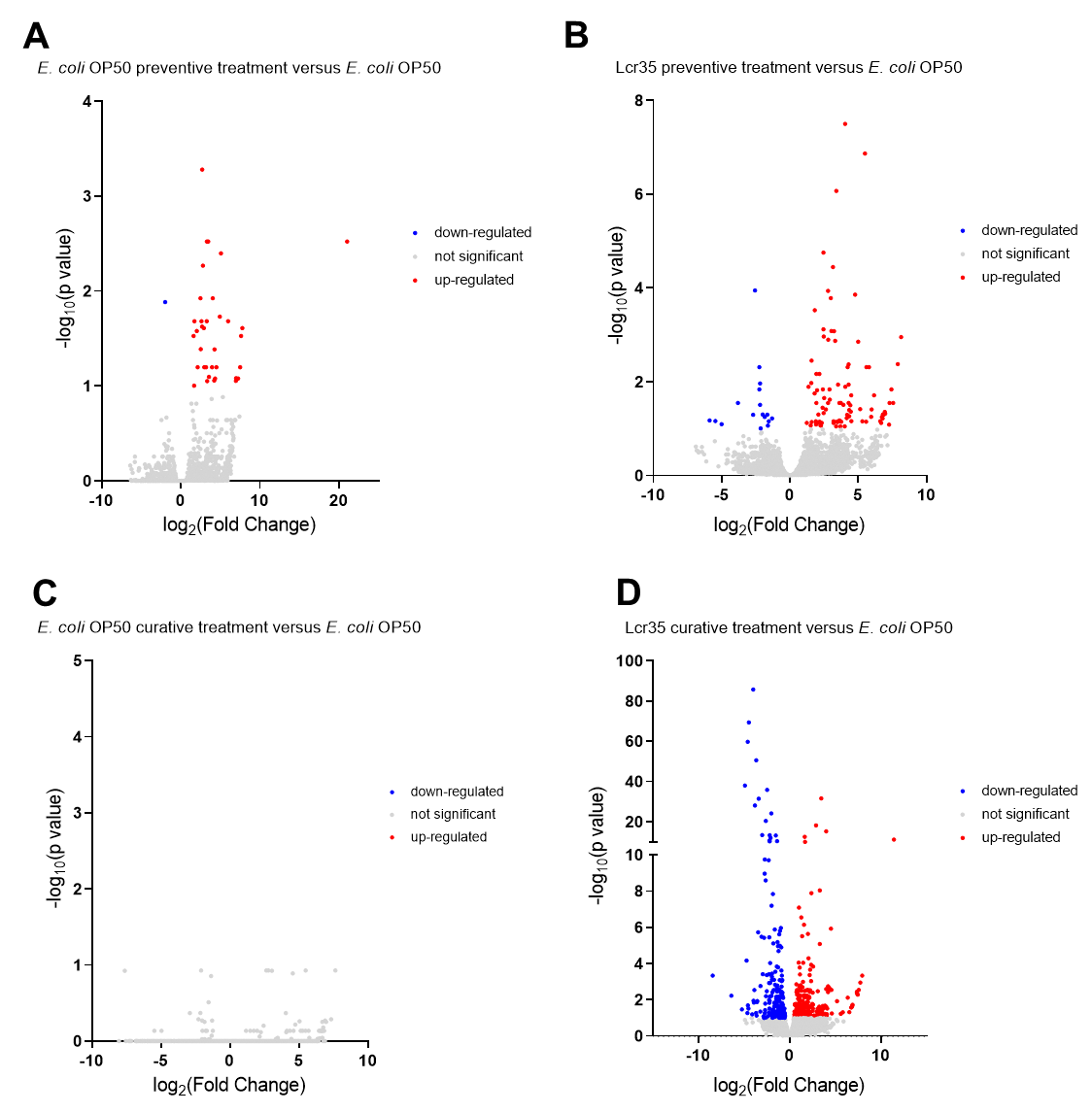


Figure S2: Volcano plots displaying *C. albicans*-infected *C. elegans* differentially expressed genes during (A) an *E. coli* OP50 preventive treatment, (B) a Lcr35^®^ preventive treatment, (C) an *E. coli* OP50 curative treatment and (D) a Lcr35® curative treatment. *C. elegans* fed with *E. coli*OP50 was used as the control condition.
